# Plant invasion alters trait composition and diversity across habitats

**DOI:** 10.1002/ece3.5130

**Published:** 2019-05-14

**Authors:** Darwin S. Sodhi, Stuart W. Livingstone, Marta Carboni, Marc W. Cadotte

**Affiliations:** ^1^ Department of Biological Sciences University of Toronto Scarborough Toronto ON Canada; ^2^ Department of Ecology and Evolutionary Biology University of Toronto Toronto ON Canada

**Keywords:** functional diversity, functional traits, habitat filtering, invasion impact, plant invasion

## Abstract

Increased globalization has accelerated the movement of species around the world. Many of these nonnative species have the potential to profoundly alter ecosystems. The mechanisms underpinning this impact are often poorly understood, and traits are often overlooked when trying to understand and predict the impacts of species invasions on communities. We conducted an observational field experiment in Canada's first National Urban Park, where we collected trait data for seven different functional traits (height, stem width, specific leaf area, leaf percent nitrogen, and leaf percent carbon) across an abundance gradient of the invasive *Vincetoxicum rossicum* in open meadow and understory habitats. We assessed invasion impacts on communities, and associated mechanisms, by examining three complementary functional trait measures: community‐weighted mean, range of trait values, and species’ distances to the invader in trait space. We found that *V. rossicum* invasion significantly altered the functional structure of herbaceous plant communities. In both habitats *V. rossicum* changed the community‐weighted means, causing invaded communities to become increasingly similar in their functional structure. In addition, *V. rossicum *also reduced the trait ranges for a majority of traits indicating that species are being deterministically excluded in invaded communities. Further, we observed different trends in the meadow and understory habitats: In the understory, resident species that were more similar to *V. rossicum* in multivariate trait space were excluded more, however this was not the case in the meadow habitat. This suggests that *V. rossicum* alters communities uniquely in each habitat, in part by creating a filter in which only certain resident species are able to persist. This filtering process causes a nonrandom reduction in species' abundances, which in turn would be expected to alter how the invaded ecosystems function. Using trait‐based frameworks leads to better understanding and prediction of invasion impacts. This novel framework can also be used in restoration practices to understand how invasion impacts communities and to reassemble communities after invasive species management.

## 1 INTRODUCTION

Many of the world's most important and valuable ecosystems have been drastically altered by invasive species (Simberloff & Rejmanek, 2011). To understand the ecological dynamics associated with these alterations, it is vital to examine both the differences between the dominant invaders and resident communities and the impacts that invaders have on them (Cadotte, Campbell, Li, Sodhi, & Mandrak, 2018; MacDougall, Gilbert, & Levine, 2009). Trait‐based analyses can provide insights into both of these aspects (Drenovsky et al., 2012).

Trait‐based analyses are increasingly being used in invasion studies because they can illustrate how invasive and native species differ in fitness and niche requirements (Funk, Standish, Stock, & Valladares, 2016). Invasive species are thought to be successful in their introduced environments because they occupy novel or empty ecological niches, and/or they possess fitness differences that drive competitive dominance over resident communities (MacDougall et al., 2009). However, measuring both niche and fitness differences is notoriously difficult, especially when considering interactions with many species. Thus, we require surrogate measures for species’ niche and fitness differences, and functional traits provide this opportunity (Cadotte, 2017; Kraft, Godoy, & Levine, 2015; Laughlin, 2014). Violle and Jiang (2009) argue that the differences between species functional traits (i.e., morphological, physiological, biological characteristics) can be used to understand niche differences. A well‐studied example of this assumption is exhibited by the invasive plant *Centaurea solstitialis, *in California grasslands, where its deep root system allows it to access water deep in the soil that other species in the recipient community cannot (Hierro, Maron, & Callaway, 2005).

**Figure 1:**
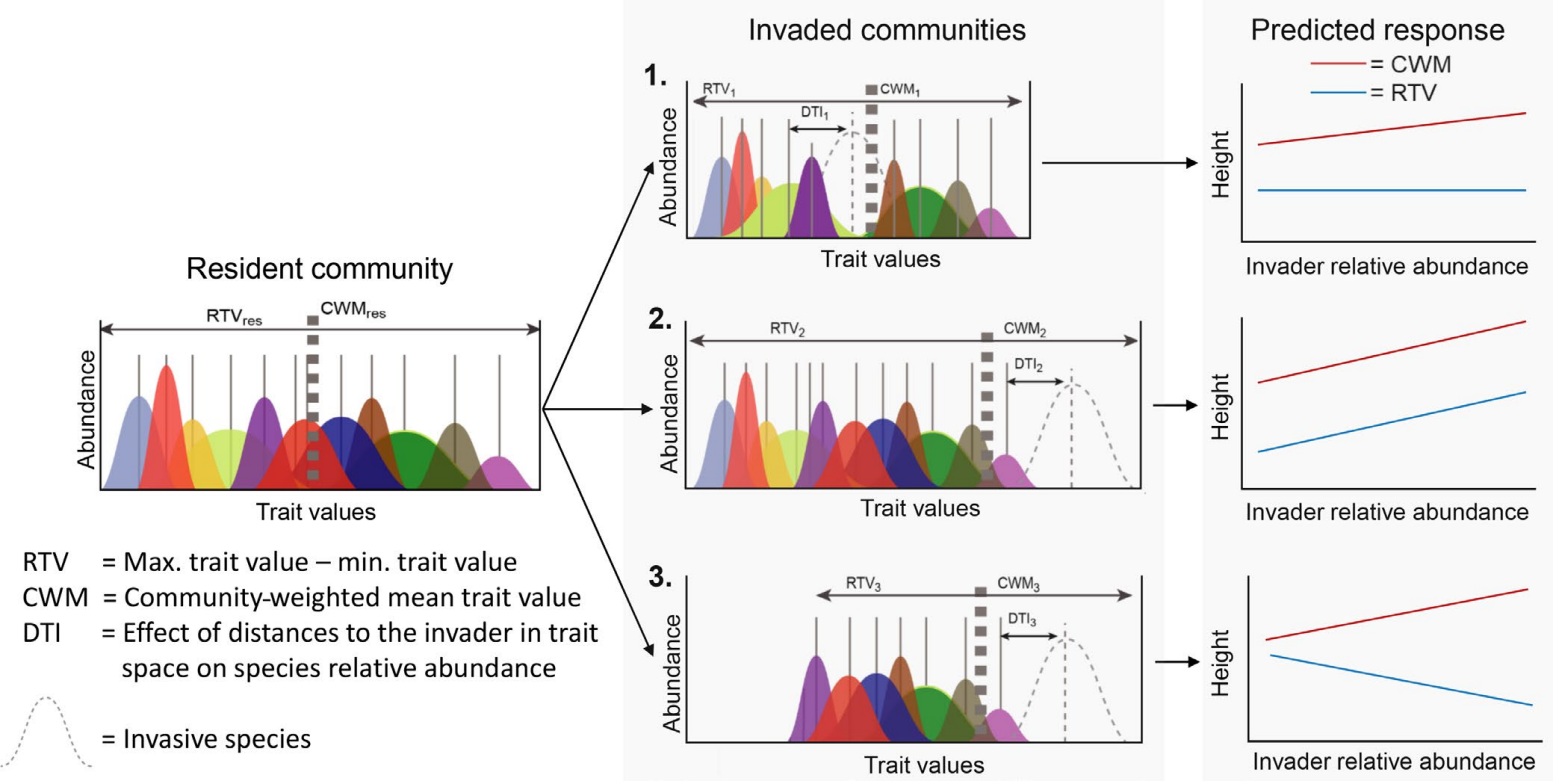
Proposed effect of an invader on resident species using three trait measures: community‐weighted means (CWM), range of trait values (RTV), and distance to invader (DTI), on resident species occurrence and relative abundance. Invaded community 1: The invader has high trait overlap with resident species. RTV of the entire community remains constant, with CWMs shifting only moderately toward the invader's own trait values, while species with low DTI are excluded. Invaded community 2: The invader occupies new niche space. RTV of the entire community increases, CWM shifts toward the trait values of the invader, and very few species have low DTI and are thus impacted through niche overlap. Invaded community 3: The invader occupies new niche space. RTV of the entire community decreases as certain species are excluded, CWM shifts toward the trait values of the invader, and very few species have low DTI and are thus impacted through niche overlap. The resulting shifts of CWM and RTV along an invasion gradient are shown on the right using height as an example for a trait

Yet, the flipside of the empty niche hypothesis is successful invaders will impact those resident species with similar resource requirements (“niche overlap hypothesis,” MacDougall et al., 2009; Gallen & Carboni, 2017). In this case, as the invader increases in abundance, we should observe decreasing abundance of species with similar traits, eventually resulting in a complete exclusion of similar species, while dissimilar species appear less impacted (MacDougall et al., 2009). In addition, invasive species have been found to cause the functional homogenization of communities across the landscape, ostensibly because they reduce diversity and eliminate certain species nonrandomly (Qian & Guo, 2010; Villéger, Grenouillet, & Brosse, 2014). These nonrandom changes in community functional diversity would be expected to alter how ecosystems function (Cadotte et al., 2018). Therefore, by examining both the functional traits of species and the manner in which functional diversity is altered during the process of invasion we gain insights not only into the possible mechanisms governing the success of invasive species, but also into the impacts of invasion. However, no single functional diversity measure captures all the relevant information to assess mechanisms for invader success and impact (e.g., Pavoine, Bonsall, Dupaix, Jacob, & Ricotta, 2017). Therefore, complementary functional diversity measures are required to fully assess invader success and impact. Here we compare three measures along an axis of increasing invader abundance, each covering specific aspects of community functional structure, relative to the invader (Figure 1): Community‐weighted mean (CWM), range of trait values (RTV), and species’ distances to the invader in trait space (DTI).

First, CWM values provide information about the contribution of dominant species to ecological processes and ecosystem function (Cadotte, 2017; Grime, 1998; Muscarella & Uriarte, 2016). Hence, on the one hand, if invaded communities show large shifts in CWM values with increasing invader abundance (converging toward the traits of the invader), this likely indicates that the invader is substantially different than the dominant species of the un‐invaded community and that the invader has occupied previously unused niche space (e.g., Invaded Community 2, in Figure 1). But, on the other hand, shifts in CWM values can also result from invasion‐driven changes in the abundances of the resident species Table S3. For example, observing an increasing CWM value for plant height across a gradient of invasion can indicate that co‐occurring species in invaded communities tend to be taller because shorter species were extirpated through shading from the competitive invader (e.g., Invaded Community 3, in Figure 1).

By examining how the range in trait values (RTV) varies across an invasion gradient, we gain insight into the influence of the invader on the total community trait space (Ordonez, Wright, & Olff, 2010). An increase in the overall RTV for a specific trait in a community following the arrival of the invasive species suggests that the invader has occupied novel trait space, lending support to the “empty niche hypothesis” (Elton, 1958; e.g., Invaded Community 2, in Figure 1). On the other hand, a decrease in RTV with increasing invasion would instead point to the creation of a strong selective biotic filter that reduces the diversity of trait values (e.g., Invaded Community 3, in Figure 1). For example, a dominant plant invader that lowers and homogenizes light availability would eliminate traits associated with shade intolerant species. This decrease in RTV is consistent with an invader acting as a selective filter that might result in community shifts not predicted by the niche overlap hypothesis.

Finally, the functional structure of an invaded community might be altered by invasion even in the absence of strong CWM or RTV shifts (e.g., Invaded Community 1, in Figure 1). This might be the case if community impact is driven mainly by niche overlap between the invader and the resident community, rather than by the introduction of novel traits (e.g., Invaded Community 2, in Figure 1) or by the creation of a selective filter (e.g., Invaded Community 3, in Figure 1). By assessing the effect of DTI on the abundance of individual species, we can test whether invasion results in the specific exclusion of species that share similar functional characteristics to the invader, and seemingly greater niche overlap. If the dominant invader is competitively superior, then we should expect that low DTI species should be most adversely affected by increasing invader abundance (MacDougall et al., 2009). However, if the invader modifies the environment in such a way that filters against the traits of certain species, then perhaps there will be no strong relationship with DTI.

We apply this framework to study how the invasive Eurasian vine *Vincetoxicum rossicum* (locally known as “dog‐strangling vine”) affects herbaceous communities in Rouge National Urban Park in Toronto, Canada. *V. rossicum* has been spreading through the Park for approximately 60 years (Moore, 1959) and is now the most dominant herbaceous plant in the Park (Livingstone, 2018). Due to its prolific rate of spread and the fact that it forms dense stands, it poses a significant threat to native biodiversity (DiTommaso, Lawlor, & Darbyshire, 2005). In addition, *V. rossicum* occurs across a number of different habitat types and at different densities in the Park. It is therefore a perfect model system to investigate how invasion alters the functional structure of plant communities in different habitats. Specifically, here we use seven plant functional traits to examine the mechanisms driving *V. rossicum* invasion and impact in two habitat types: open meadows and forest understory.

We predict that *V. rossicum* will alter invaded communities, both by dominating the functional structure of the community and by selectively excluding species with particular trait values through the creation of a filter (e.g., short species excluded because of shading). Previously conducted meta‐analyses and experimental work have shown that invasive plants tend to have higher trait values for height, stem width, specific leaf area (SLA), leaf nitrogen content (LNC), and leaf carbon content (LCC), while having lower trait values in leaf dry matter content (LDMC) and number of leaves compared to resident species in invaded communities (Van Kleunen, Dawson, & Dostál, 2011; Jakobs, Weber, & Edwards, 2004; Liao et al., 2008). Based on this, we expect that increasing *V. rossicum* abundance will be positively correlated with CWM values for height, stem width, SLA, LNC, and LCC, and negatively correlated with number of leaves and LDMC. Secondly, we predict that increasing abundance of *V. rossicum* will lead to a decrease in the RTV in the community, which would indicate that the invader nonrandomly excludes certain resident species by altering local environmental conditions. However, there can be CWM shifts without increases to RTV if the invader replaces species with similar traits (e.g., Invaded Community 1, in Figure 1), or it occupies unique space while excluding dissimilar species (e.g., Invaded Community 3, in Figure 1). In order to tease apart this effect, we contrasted two RTV measures for invaded communities; one including *V. rossicum*'s values and the other excluding them. Finally, if *V. rossicum* competes most with similar species, we expect that resident species that are further away from the invader in functional trait space (greater DTI values) will be unaffected in invaded communities, while resident species with traits closer to the invader will be more likely to be outcompeted.

## 2 METHODS

### 2.1 Site

The observational field study was conducted in Rouge National Urban Park, located in Toronto, Ontario, Canada's largest city. This study was conducted in the summer of 2016, across 23 sites in two distinct habitats: meadow (open, sunny areas) and forest understory (shaded areas). We set 13, 50 by 50 m sites in meadow habitat and 10, 30 by 30 m sites in understory habitat. Each site was stratified into an equidistant grid of 25 plots, totalling 575 plots for the full study. Sites were chosen based on a varying degree of *V. rossicum* abundance (i.e., our invasion gradient). To quantify species abundances, two trained observers estimated the two‐dimensional area occupied by each species’ in each of the study plots to attain a value of percent cover. The meadow habitat included a total of 31 resident species for which we were able to obtain trait data, out of which 15 species are exotic. The understory habitat included a total of five resident species for which we were able to obtain trait data, out of which three species are exotic.

### 2.2 Field sampling and laboratory processing

In summer 2016, from early June until mid‐October, data for seven traits were collected from the 23 sites from 36 species across the two habitats: height, stem width, number of leaves, SLA, LDMC, LNC, and LCC. Each of these traits has been shown to predict plant strategy and competitive ability (Table 1). For each sampled species: height, stem width, and number of leaves were measured up to a maximum of 20 individuals and a minimum of 5 individuals per site. For each sampled species, trait measurements were taken at peak flowering time. The individuals sampled were collected throughout the sites. Plant height was measured from the bottom of the stem to the highest foliage using a meter ruler (Perez‐Harguindeguy et al., 2013). Stem width was taken at the base of the stem and measured using Neiko digital calipers, which were accurate to three significant figures (Perez‐Harguindeguy et al., 2013). Number of leaves were counted using numerators (Perez‐Harguindeguy et al., 2013). Two leaf samples that were unmarked by insect or pathogen damage were collected from the top half of plants from all species present, up to a maximum number of 20 individuals per site (Perez‐Harguindeguy et al., 2013). This resulted in the collection of 3,587 leaves from 43 species.

**Table 1:** Synthesis of traits and functions recorded for plant species in the Rouge National Urban Park during 2016 field season

Trait	Abbreviation	Correlation to plant strategy/function (Kleyer et al., 2008; Perez‐Harguindeguy et al., 2013)
Height	N/A	Competitive vigor, reproductive size, fecundity, potential lifespan, and resilience
Specific leaf area	SLA	Positively associated with relative growth rate (RGR), photosynthetic rate, and leaf nitrogen content
Leaf dry matter content	LDMC	Negatively associated with RGR and positively associated with leaf lifespan
Leaf percent carbon	LCC	Carbon inputs into soil, herbivory rates and biomass production
Stem width	N/A	Stability, defense, architecture, carbon gain and growth potential
Number of leaves	N/A	Competitive strategy of the plant and longevity of leaves
Leaf percent nitrogen	LNC	Photosynthetic rate positively associated with RGR

Note

Bold values indicate statistical significance (p<0.05).

Leaf samples were frozen at −20°C for at least 24 hr (Kleyer et al., 2008). After leaves were thawed in deionized water, the fresh weight was measured, and leaves were scanned to determine leaf area (Kleyer et al., 2008). The leaves were then dried for a minimum of 48‐hr, in a VWR standing oven 70°C and then reweighed for dry weight. All the weighing took place using a Mettler Toledo ML Series precision balance. Specific leaf area (SLA) was calculated as area of a leaf in millimeters squared (mm^2^) divided by the dry weight of the same leaf in milligrams (mg). Leaf dry matter content (LDMC) was calculated as the dry weight of a leaf in mg divided by the fresh weight of the same leaf in grams.

Leaf nitrogen content and leaf carbon content were determined using the LECO 628 series elemental analyzer. Composite samples were made for species with extremely small leaves using leaves collected from the same plots, as the minimum weight that the elemental analyzer can detect is 0.1 g.

### 2.3 Trait‐based analyses

We calculated three trait‐based metrics: (a) community‐weighted mean trait values (CWM), (b) range of trait values (RTV), and (c) distance to invader in trait space (DTI). For all analyses, a community refers to a single experimental plot within the sites. All trait‐based metrics, unless otherwise stated, included the traits of the invader, *V. rossicum* Table S3.

Community‐weighted mean values were calculated for each trait by multiplying the mean trait value (*t*) of species *i *across all plots by the proportional abundance using cover estimates (a) of species *i *in plot *j*:

$${\text{CWM}}_{i,j}=\sum_{i=1}^{S_{j}}\frac{a_{i,j}\cdot t_{j}}{\sum_{i=1}^{S_{j}}a_{i,j}}$$

Range of trait values was calculated for each trait by taking the maximum trait value and subtracting the minimum value to obtain a trait range value in each plot. Trait values used were site averages for each species present in the plots (Luo et al., 2016). For the meadow sites, two RTVs were calculated, in one case by including and in the other by excluding *V. rossicum* trait values, in order to disentangle the effect, the invader had on trait space. However, this double analysis could not be conducted with understory plots since many of the invaded plots contained very few species other than *V. rossicum*, so for the understory habitat RTV was only calculated including the invader.

As a preliminary step, in order to assess whether the variance in CWM and RTV across plots was constant along the invasion gradient, we grouped plots into four groups corresponding to increasing abundances of *V. rossicum* (e.g., 0–0.25 and 0.25–0.5) and then ran Levene's homogeneity of variance test across the groups. With this analysis we also aimed to screen for potential homogenization (i.e., lower variance across plots) in the functional structure of communities at high *V. rossicum* abundance.

We then used linear mixed effect models to test whether CWMs and RTV were affected by *V. rossicum* abundance. Both trait‐based measures were used as dependent variables in the models with *V. rossicum* relative abundance as the independent variable and site as a random factor. We used the percent cover data to estimate *V. rossicum *relative abundance in each plot. Separate models were fit for the understory and meadow sites. All statistical analyses were carried out using R statistical software (R Core Team, 2015). The dbFD package was used to calculate measures of community‐weighted means (Laliberté & Legendre, 2010). LME4 package was used to fit the linear mixed effect models (Bates, Maechler, Bolker, & Walker, 2015). The car package was used to calculate Levene's test (Fox & Weisberg, 2011).

Finally, to assess the effect of DTI on co‐occurring species abundances, we followed two steps: (a) estimating the effect of the invader on each species and (b) relating this effect to the functional distance to the invader (DTI). In the first step, we used the lme4 package (Bates et al., 2015) to fit, for each species, linear mixed effects models, with site set as a random factor, to examine the relationship between the relative abundances of all other species in each community and the relative abundance of *V. rossicum*. This was done to quantify the effect that the invader has on each species in this experiment. Negative relationships indicate that species decrease in abundance with increasing invader abundance, while positive values indicate the opposite. In the second step, we then treated the slope coefficients of each of these species‐level models as the response variable in linear regressions (one for the understory and one for the meadow) with the functional distance to *V. rossicum* (DTI) as the predictor variable. To obtain DTI, we first calculated a pairwise species functional distance matrix which included all seven traits recorded for all species in each habitat using the Funrar package in R (Grenié, Denelle, Tucker, Munoz, & Violle, 2017). Gower's distance was used to combine all traits recorded into a single multivariate functional distance measure. From this matrix, we extracted the distance between each resident species in the species pool and *V. rossicum*. A positive relationship of the invader effect on abundance to DTI indicates that species that are less functionally distant from DSV tend to be more negatively affected by invasion.

## 3 RESULTS

### 3.1 Community‐weighted mean (CWM) trait values

#### 3.1.1 Meadow habitat

**Figure 2:**
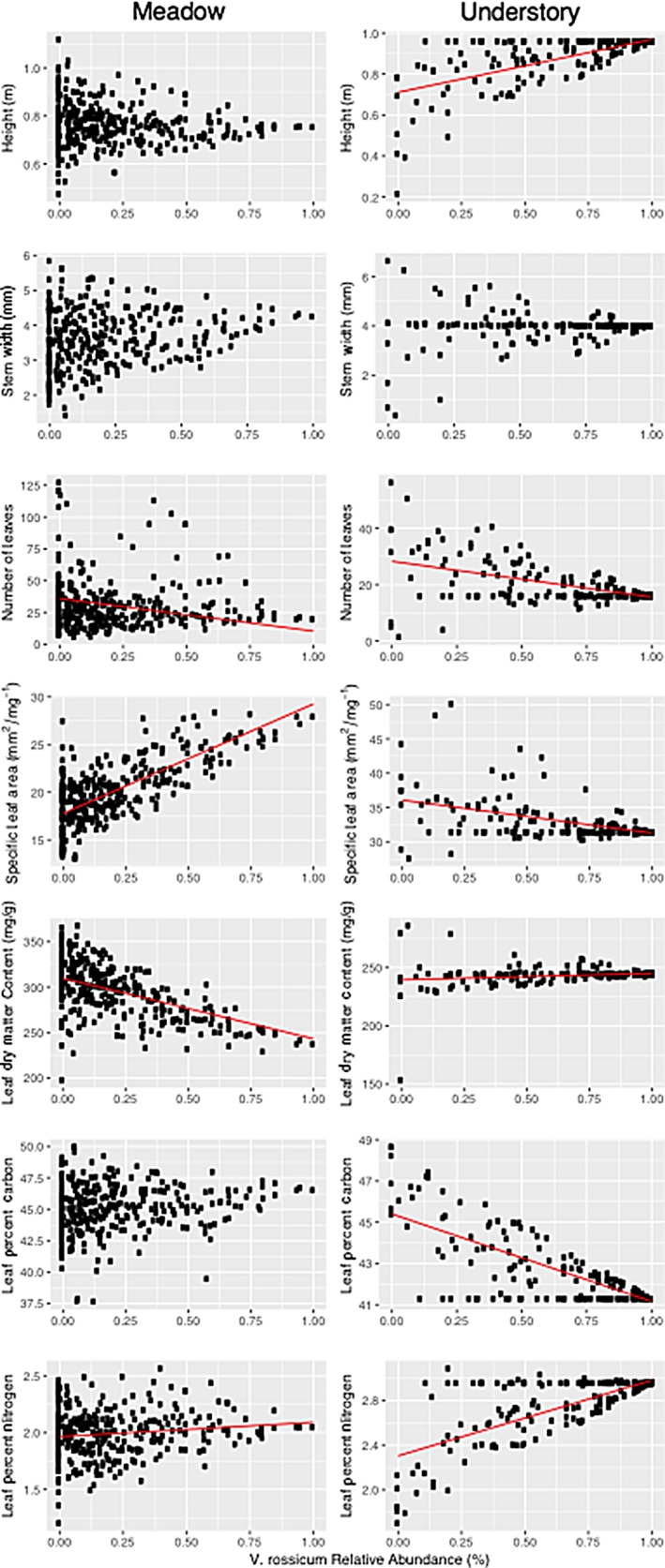
The relationship between community‐weighted mean and *V*. *rossicum* relative abundance for seven functional traits. Red lines are shown for significant relationships (*p*‐value < 0.05). Meadow Height: *R* ^2^ = 0.001, *t*_322_ = −0.43, *p* = 0.665. Meadow Stem Width: *R* ^2^ = 0.01, *t*_322_ = 1.55, *p* = 0.122. Meadow Number of Leaves: *R* ^2^ = 0.066, *t*_322_ = −4.54, *p *≤ 0.001. Meadow Specific Leaf Area: *R* ^2^ = 0.548, *t*_322_ = 17.3, *p *≤ 0.001. Meadow Leaf Dry Matter Content: *R* ^2^ = 0.266, *t*_322_ = −9.88, *p *≤ 0.001. Meadow Leaf Percent Carbon: *R* ^2^ = 0.001, *t*_322_ = −0.20, *p* = 0.841. Meadow Leaf Percent Nitrogen: *R* ^2^ = 0.018, *t*_322_ = 2.02, *p* = 0.045. Understory Height: *R* ^2^ = 0.502, *t*_249_ = 15.8, *p *≤ 0.001. Understory Stem Width: *R* ^2^ = 0.010, *t*_249_ = 1.58, *p* = 0.116. Understory Number of Leaves: *R* ^2^ = 0.312, *t*_249_ = −10.6, *p *≤ 0.001. Understory Specific Leaf Area: *R* ^2^ = 0.279, *t*_249_ = −9.77, *p *≤ 0.001. Understory Leaf Dry Matter Content: *R* ^2^ = 0.039, *t*_249_ = −3.19, *p* = 0.002. Understory Leaf Percent Carbon: *R* ^2^ = 0.621, *t*_249_ = −20.0, *p *≤ 0.001. Understory Leaf Percent Nitrogen: *R* ^2^ = 0.577, *t*_249_ = 18.3, *p *≤ 0.001

In the meadow habitat, number of leaves, SLA, LDMC, and LNC were all significantly related to *V. rossicum* relative abundance in the community (Figure 2). Specifically, in accordance with our predictions, number of leaves and LDMC were negatively correlated, while SLA and LNC were positively correlated with *V. rossicum* abundance (Table 2). In contrast to our predictions, we found no significant relationship of height with *V. rossicum* abundance in this habitat. Levene's test showed that variances for all traits, except # of Leaves an SLA, were unequal across invader abundance groupings, and specifically decreased with increasing *V. rossicum* abundance (*p*‐value < 0.05, Table S1).

**Table 2:** Linear mixed effect models of CWM values in the Meadow and Understory habitats as a function of invader abundance

Response variable	Estimate	*SE*	Marginal *R* ^2^	Conditional *R* ^2^	*t*‐value	*df*	*p*‐value
Meadow							
Height	−0.012	0.027	0.001	0.209	−0.43	322	0.665
Stem width	0.370	0.239	0.01	0.254	1.55	322	0.122
# Leaves	**−25.5**	**5.62**	**0.066**	**0.474**	**−4.54**	**322**	**<0.001**
SLA	**11.5**	**0.662**	**0.548**	**0.687**	**17.3**	**322**	**<0.001**
LDMC	**−66.6**	**6.74**	**0.266**	**0.542**	**−9.88**	**322**	**<0.001**
LCC	−0.111	0.555	0.001	0.359	−0.20	322	0.841
LNC	**0.127**	**0.063**	**0.018**	**0.195**	**2.02**	**322**	**0.045**
Understory							
Height	**0.259**	**0.016**	**0.502**	**0.533**	**15.8**	**249**	**<0.001**
Stem width	0.194	0.125	0.010	0.118	1.58	249	0.116
# Leaves	**−12.7**	**1.20**	**0.312**	**0.349**	**−10.6**	**249**	**<0.001**
SLA	**−4.81**	**0.491**	**0.279**	**0.323**	**−9.77**	**249**	**<0.001**
LDMC	**5.56**	**1.75**	**0.039**	**0.042**	**−3.19**	**249**	**0.002**
LCC	**−4.24**	**0.212**	**0.621**	**0.637**	**−20.0**	**249**	**<0.001**
LNC	**0.676**	**0.036**	**0.577**	**0.603**	**18.3**	**249**	**<0.001**

Predictor variable was the relative abundance of *V. rossicum*, site was included as a random effect.

Bold values indicate statistical significance (*p* < 0.05).

#### 3.1.2 Understory habitat

In the understory habitat, CWM values for all traits, except stem width, were significantly explained by *V. rossicum* relative abundance (Figure 2). Models for height, LDMC, and LNC showed positive correlations to invader abundance (Table 2). However, models for number of leaves, SLA, and LNC showed negative correlations to *V. rossicum*, which was partially in contrast to our a priori predictions (Table 2). Again, Levene's test showed that variances for all traits were unequal across invasion abundance groupings and specifically increased with increasing *V. rossicum* abundance (*p*‐value < 0.05; Table S1).

### 3.2 Range of trait values (RTV)

#### 3.2.1 Meadow Habitat including invader trait values

**Figure 3:**
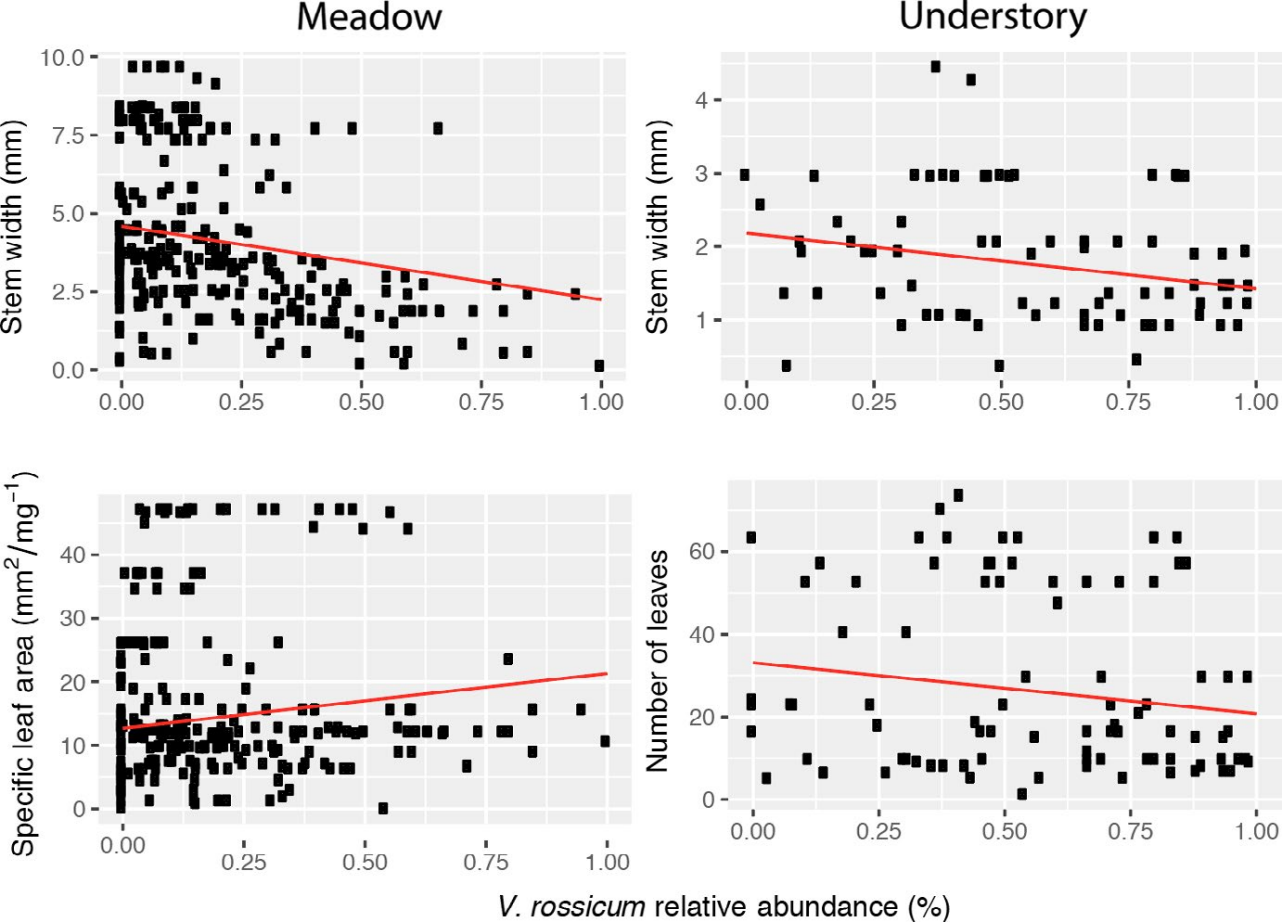
The relationship between trait range and *V. rossicum* relative abundance for stem width, specific leaf area, and number of leaves (including *V. rossicum* trait values). Red lines are shown for significant relationships (*p*‐value < 0.05). Meadow Stem Width: *R* ^2^ = 0.043, *t*_276_ = −3.39, *p *≤ 0.001. Meadow Specific Leaf Area: *R* ^2^ = 0.021, *t*_260_ = 2.83, *p* = 0.005. Understory Stem Width: *R* ^2^ = 0.052, *t*_77_ = −2.95, *p* = 0.004. Understory Number of Leaves: *R* ^2^ = 0.025, *t*_89_ = −2.25, *p* = 0.027

For the analyses conducted in the meadow that included *V. rossicum* trait values, height, stem width, and number of leaves were negatively correlated with invader relative abundance while SLA was positively correlated with invader relative abundance (Figure 3). However, these relationships were statistically significant only for stem width and SLA (Figure 3). Levene's test showed variance for all traits execept SLA and LNC were unequal across *V.rossicum* abundance.

#### 3.2.2 Meadow Habitat excluding invader trait values

**Figure 4:**
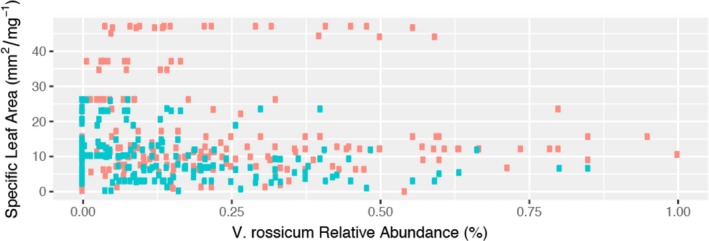
The relationship between trait range in the meadow including and excluding *Vincetoxicum rossicum* values for specific leaf area. There is a statistically significant difference (*p*‐value < 0.001) between values of RTVs for SLA if *V. rossicum* trait values are included (red) or excluded (blue). Points have been jittered

When we excluded *V. rossicum* trait values in the RTV calculation, all trait ranges decreased with increasing *V. rossicum* abundance, though none of these relationships were statistically significant (only marginally significant for height, # leaves and LCC; Table 3). In addition, comparing the two RTV values (Figure 4) using a Student *t* test showed that in the case of SLA there was a statistically significant difference in the mean RTV value calculated with or without the invader (*p*‐value < 0.001). Levene's test showed that variances for height, stem width and # of leaves were unequal across *V. rossicum* abundance groupings and specifically decreased with increasing invader abundance (*p*‐value < 0.05; Table S2).

**Table 3:** Linear mixed effect models of RTV in the meadow (including & not including invader traits values) and understory habitat

Response variable	Estimate	*SE*	Marginal *R* ^2^	Conditional *R* ^2^	*t*‐value	*df*	*p*‐value
Meadow (Including Invader trait values)							
Height	−0.124	0.063	0.008	0.742	−1.95	276	0.052
Stem width	**−2.35**	**0.692**	**0.043**	**0.512**	**−3.39**	**276**	**<0.001**
# Leaves	−25.4	13.8	0.014	0.470	−1.84	276	0.067
SLA	**8.61**	**3.04**	**0.021**	**0.687**	**2.83**	**260**	**0.005**
LDMC	9.36	17	<0.001	0.765	0.550	260	0.583
LCC	−0.843	0.792	0.005	0.402	−1.06	264	0.289
LNC	0.058	0.176	<0.001	0.663	0.331	264	0.741
Meadow (Excluding Invader trait values)							
Height	−0.176	0.091	0.011	0.655	−1.93	215	0.055
Stem width	−0.979	0.857	0.005	0.582	−1.14	215	0.255
# leaves	−35.6	19.1	0.016	0.411	−1.86	215	0.065
SLA	−3.26	2.10	0.006	0.693	−1.55	192	0.124
LDMC	−14.5	21.8	0.001	0.712	−0.663	192	0.508
LCC	−1.80	0.972	0.018	0.359	−1.85	201	0.066
LNC	−0.3	0.198	0.008	0.579	−1.51	201	0.132
Understory							
Height	−0.006	0.076	<0.001	<0.001	−0.078	89	0.939
Stem width	**−0.756**	**0.257**	**0.052**	**0.630**	**−2.95**	**77**	**0.004**
# Leaves	**−12.3**	**5.46**	**0.025**	**0.647**	**−2.25**	**89**	**0.027**
SLA	−2.65	2.27	0.003	0.851	−1.17	85	0.245
LDMC	−24	15.7	0.015	0.547	−1.51	85	0.133
LCC	0.234	0.206	0.003	0.836	1.14	85	0.258
LNC	−0.120	0.165	0.012	0.451	−1.21	85	0.229

Note

Predictor variable was the relative abundance of *V. rossicum*, site was included as a random effect.

Bold values indicate statistical significance (*p* < 0.05).

#### 3.2.3 Understory Habitat

In the understory, only models of stem width and number of leaves exhibited statistically significant relationships of RTVs with *V. rossicum* abundance (Figure 3). However, for all traits except LCC, there were negative correlations to *V. rossicum* relative abundance, which indicates that as invader relative abundance increases trait ranges generally decrease (Table 3). Levene's test showed that variance for stem width across the gradient in *V. rossicum* abundance was unequal, and specifically, variance showed a bell shape distribution (*p*‐value < 0.05; Table S2).

#### 3.2.4 Effect of distances to invader on species relative abundances

**Figure 5:**
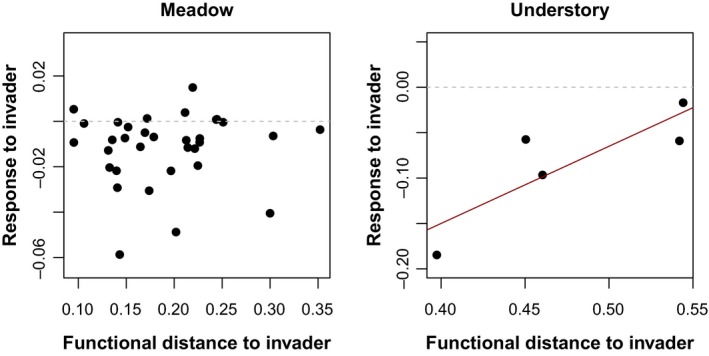
Relationship between the abundance response of species to invasion and distances to the invader in functional trait space in the meadow (left panel) and understory (right panel). Meadow: *R* ^2^ = 0.00151, *F*_32_ = 0.0483, *p* = 0.827. Understory: *R* ^2^ = 0.730, *F*_3_ = 7.70, *p* = 0.069

In the meadow habitat, we found a nonsignificant negative relationship between DTI and the coefficients of species relative abundance versus invader abundance (Figure 5).

On the contrary, in the understory we found a marginally significant positive relationship between DTI and how species relative abundance was affected by *V. rossicum *(Figure 5). This indicates that the abundance of species further away from *V. rossicum* in functional trait space tends to be unaffected in highly invaded communities, while species with traits similar to the invader tend to decrease in abundance.

## 4 DISCUSSION

The results of this study indicate that invasion by *V. rossicum* significantly alters the trait composition of herbaceous plant communities, in both meadow and understory habitats. Our specific predictions about how *V. rossicum* would change the CWM of traits in invaded communities were partially supported for certain traits in both the meadow and understory. The second prediction, that *V. rossicum* would decrease RTV in both habitat types, was again partially supported in the meadow and the understory habitat for certain traits. A positive relationship between RTVs and *V. rossicum* abundance was only found for SLA in the meadow and was the same when including or excluding *V. rossicum* trait values in the RTV calculation, which supports the hypothesis that *V. rossicum* makes a novel contribution to trait space for SLA in this habitat. Finally, the last prediction that relative abundances of co‐occurring species would decrease if they had lower DTI values was supported in the understory but not in the meadow.

Overall, our results indicate that *V. rossicum* creates a filter that alters communities deterministically, though in different ways across habitats. In the meadow, when *V. rossicum* abundance increases, the resulting community consists of faster growing species with higher LNC. But the species that are present in these invaded communities also have fewer leaves, with greater water content compared to less invaded sites. However, in the understory, when *V. rossicum* abundance increases, the resulting community is taller on average with lower water content and greater nitrogen content in the leaves. In addition, the more invaded understory communities have lower number of leaves, with slower growing species that have low LCC. These traits are those observed in *V. rossicum* itself, which suggests that CWM shifts are determined at least partially by *V. rossicum* inputting novel functions in the community, which is in line with the empty niche hypothesis (MacDougall et al., 2009). Our results are similar to Hejda and De Bello, (2013), and Knapp and Kühn, (2012), which have shown that biological invaders were functionally dissimilar to resident species co‐occurring in a habitat and that this is a mechanism through which invasions can alter communities.

Second, we found that in the meadow, species richness is reduced by six species in the maximally invaded plots relative to un‐invaded plots. Similarly, in the understory species richness is reduced by four species in the fully invaded plots. One consequence of this diversity loss is that the RTV decreases for a number of traits with increasing abundance of *V. rossicum*, indicating that certain resident species are excluded based on their functional characteristics. In the meadow habitat, trait ranges in the community were reduced for height, stem width, and number of leaves, while in the understory only RTV for stem width and number of leaves were reduced. This result highlights that *V. rossicum* is partially determining which species are able to persist in both habitats, though this effect is stronger in the meadow. Specifically, the reduction in RTV for height potentially indicates that *V. rossicum* has a greater competitive ability (or fitness advantage) for light interception compared to the resident community.

**Figure 6:**
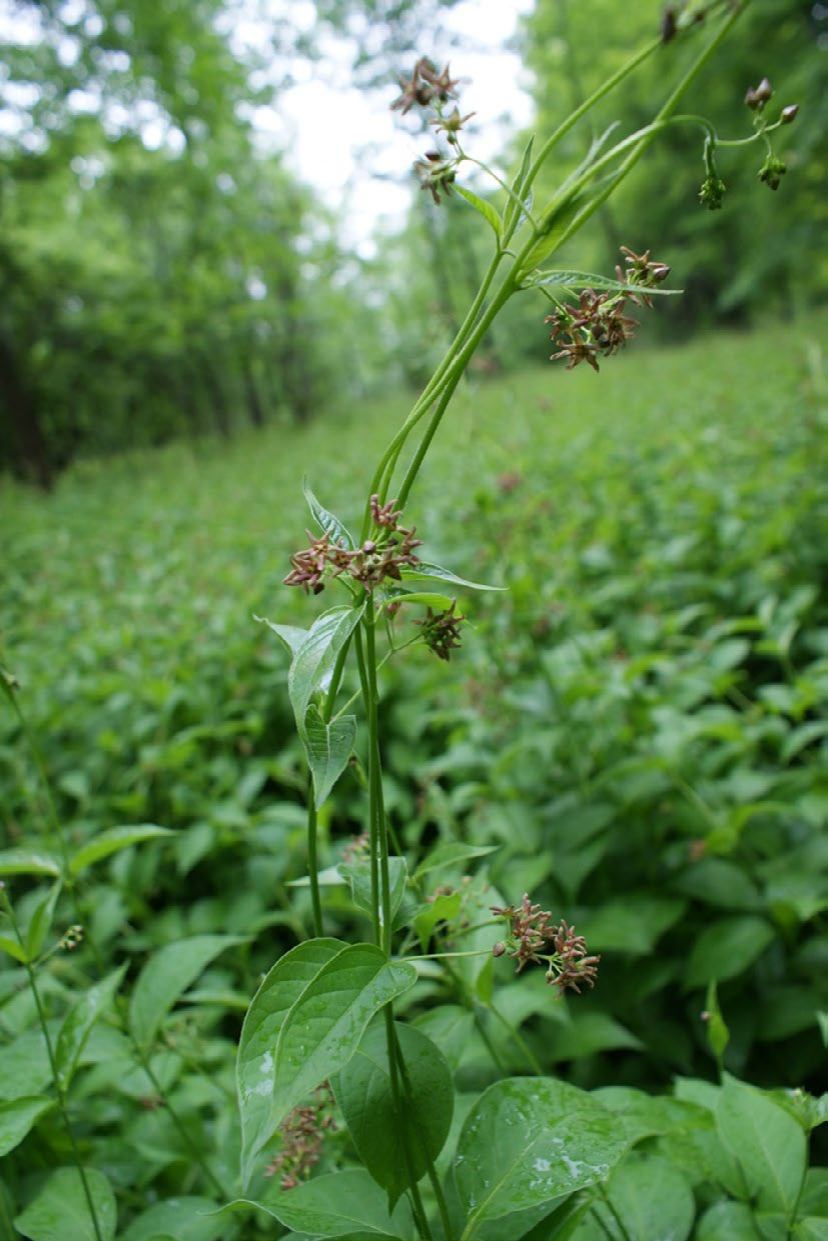
*Vincetoxicum rossicum *at an understory study site in the Rouge National Urban Park in Toronto, Ontario, Canada. Photograph: m. Cadotte

Differences that arise between the two habitats can be understood by considering the different environmental conditions. In the understory, species are already adapted to low light, so *V. rossicum* does not introduce a strong new filter for this resource. However, *V. rossicum* is likely to shade meadow species selecting for taller species that can still access light in the presence of *V. rossicum,* leading to the observed lower RTV for height (given that height is related to competitive ability for light, Pérez‐Harguindeguy et al., 2013; Moles et al., 2009). However, RTV for SLA increased with invader abundance in the meadow, indicating that CWM shifts for this trait are driven by *V. rossicum* dominance rather than by the displacement of certain resident species (e.g., Invaded Community 2 in, Figure 6).

Indeed, by comparing RTV of the invaded communities between the analyses that included and excluded the invader, it is clear that the invader makes a novel contribution to SLA trait space in the meadow habitat (i.e., empty niche hypothesis). This result highlights that *V. rossicum* possesses a much higher SLA than most species of un‐invaded meadow communities, which, in turn, suggests that *V. rossicum* has a higher relative growth rate (RGR) compared to resident species (Perez‐Harguindeguy et al., 2013). Further, this result is in line with studies by Grotkopp, Rejmánek, and Rost (2002) and Grotkopp and Rejmánek, (2007) who highlighted that SLA and RGR are very important traits that determine invasiveness in plant species.

The fact that the invader changed the community in different ways in the two habitats was also apparent by examining how DTI values related to species relative abundances. In the understory habitat, species that were further away from *V. rossicum* in functional trait space were less affected by invasion compared to species closer to the invader in functional trait space. However, this was not the case in the meadow habitat, where the same analysis showed no discernable trend. This suggests that niche overlap, as opposed to differences in competitive ability for light, is a more important mechanism of invasion impact in the understory than in the meadow. As a consequence, in the understory, species that overlap too much in their traits with *V. rossicum* tend to be excluded in highly invaded communities, while species that are functionally different are less affected (e.g., Invaded Community 1, in Figure 1). Nevertheless, on a whole, *V. rossicum* reduced the abundance of every other species in the understory, which is consistent with the idea that the invader has a higher fitness compared to most resident species (MacDougall et al., 2009).

### 4.1 Broader implications

The results of this study demonstrate how an invasive species can change communities through alterations to community functional structure. In addition, to communities being increasingly dominated by the traits of the invader, certain species and their traits, are persisting with apparently little negative impact, while other species are excluded from communities. As a consequence, the variance in functional structure (regardless of the exact measure) across communities decreases across the invasion gradient. The net result of these functional alterations is that invaded communities become increasingly spatially homogeneous in their traits and less functionally diverse. This systematic functional alteration will likely result in changes to ecosystems function (Cadotte, Carscadden, & Mirotchnick, 2011). Differing trends across habitats also highlights the need to consider both environmental and biotic filters as factors in the process of invasion‐driven community change.

Our finding that an invader can act as a filter, causing trait shifts in invaded communities mirrors similar trends being observed for other invasive species in different habitats (Gallien & Carboni, 2017). Furthermore, the clear fitness advantage of *V. rossicum* over resident communities also points to a typical mechanism through which invasive species are more successful than resident communities. However, it also needs to be noted that some of these trends could be the results of differential site histories and characteristics. This is because, though we did include site as random factor in our models, the effect of site histories was not included explicitly in the analyses and it is known to be a potential confounding factor in biological invasions (Ehrenfeld, 2010).

Finally, we also found that *V. rossicum* is more prevalent in the understory, which could be because of lower species richness in this habitat. Species richness has been shown to reduce the effects of biological invasions in previous studies by reducing the rate of establishment and in certain cases repelling invasion all together (Fargoine & Tilman, 2005). This trend indicates support of the diversity‐resistance hypothesis, which states that in more diverse communities there is increased competition for niche space, and this acts as a barrier to potential invaders (Levine, Antonio, Levine, & Antonio, 2010). Conducting experiments on biological invaders in two distinct habitats can provide insights into how invasive species dynamics and impacts can vary across distinct landscapes. Trait‐based assessments to quantify impacts of invasive species are seldom used in invasion ecology, however they provide valuable insights into underlying mechanisms and impacts that aren't entirely observable using other methods. Further, by using trait‐based analyses to characterize how communities are impacted during invasion, we gain insight into which traits, and their diversity, need to be considered when restoring invaded ecosystems (Laughlin, 2014; Ostertag, 2015). Overall using trait‐based frameworks such as the one used in this study allows for a more complete and nuanced understanding of how invasive species impact communities.

## AUTHOR CONTRIBUTIONS

MWC and DSS conceived the ideas and designed methodology; DSS and SWL collected the data; DSS and SWL analyzed the data; DSS and MC led the writing of the manuscript. All authors contributed critically to the drafts and gave final approval for publication.

## Data Availability

Data from this study has been submitted to DRYAD. Data package title: Data from: Plant invasion alters trait composition and diversity across habitats. Journal: Ecology and Evolution. Provisional DOI: https://doi.org/10.5061/dryad.6rn879c. Data files: Sodhi et al 2019 ‐ Trait & abundance data.
