# Supplementary material for: Plant invasion alters trait composition and diversity across habitats
Source: Ecol Evol. 2019 May 14;9(11):6199–210. doi: 10.1002/ece3.5130 (PMC6580280; doi:10.1002/ece3.5130)
Supplement: Supplementary file 1 [file ECE3-9-6199-s001.docx]

Supporting Information

Table 1:Levene’s test results for CWM values in the Meadow and Understory habitats. Factor defining groups was DSV relative abundance split into quarters (0-0.25, 0.25-0.5, 0.5-0.75, 0.75-1).

| **Response Variable** | **F-value** | **df** | **p-value** |
| --- | --- | --- | --- |
| **Meadow** |  |  |  |
| **Height** | **9.78** | **3** | **<0.001** |
| **Stem Width** | **9.35** | **3** | **<0.001** |
| # Leaves | 1.81 | 3 | 0.144 |
| SLA | 2.20 | 3 | 0.088 |
| **LDMC** | **5.13** | **3** | **0.001** |
| **LCC** | **5.35** | **3** | **0.001** |
| **LNC** | **4.09** | **3** | **0.007** |
| **Understory** |  |  |  |
| **Height** | **86.6** | **3** | **<0.001** |
| **Stem Width** | **58.5** | **3** | **<0.001** |
| **# Leaves** | **97.8** | **3** | **<0.001** |
| **SLA** | **45.1** | **3** | **<0.001** |
| **LDMC** | **33.0** | **3** | **<0.001** |
| **LCC** | **86.8** | **3** | **<0.001** |
| **LNC** | **106** | **3** | **<0.001** |

Table 2: Levene’s test results for RTV in the Meadow (including & not including DSV traits values) and Understory habitat. Factor defining groups was DSV relative abundance split into quarters (0-0.25, 0.25-0.5, 0.5-0.75, 0.75-1).

| **Response Variable** | **F-value** | **df** | **p-value** |
| --- | --- | --- | --- |
| **Meadow (Including DSV trait values)** |  |  |  |
| **Height** | **8.15** | **3** | **<0.001** |
| **Stem Width** | **11.5** | **3** | **<0.001** |
| **# Leaves** | **6.91** | **3** | **<0.001** |
| SLA | 2.14 | 3 | 0.095 |
| **LDMC** | **4.38** | **3** | **0.005** |
| **LCC** | **7.51** | **3** | **<0.001** |
| LNC | 2.01 | 3 | 0.112 |
| **Meadow (excluding DSV trait values)** |  |  |  |
| **Height** | **5.65** | **3** | **<0.001** |
| **Stem Width** | **4.41** | **3** | **0.005** |
| **# Leaves** | **6.92** | **3** | **<0.001** |
| SLA | 2.78 | 3 | 0.043 |
| LDMC | 2.49 | 3 | 0.062 |
| LCC | 2.15 | 3 | 0.096 |
| LNC | 3.81 | 3 | 0.011 |
| **Understory** |  |  |  |
| Height | 1.46 | 3 | 0.232 |
| Stem Width | 3.75 | 3 | 0.015 |
| # Leaves | 1.81 | 3 | 0.153 |
| SLA | 2.62 | 3 | 0.058 |
| LDMC | 0.214 | 3 | 0.887 |
| LCC | 1.15 | 3 | 0.336 |
| LNC | 1.14 | 3 | 0.339 |

| **Response Variable** | **Estimate** | **Std error** | **Marginal R^2^** | **Conditional**  **R^2^** | **t-value** | **df** | **p-value** |
| --- | --- | --- | --- | --- | --- | --- | --- |
| **Meadow** |  |  |  |  |  |  |  |
| Height | -0.055 | 0.035 | 0.010 | 0.150 | -1.58 | 318 | 0.114 |
| **Stem Width** | **-0.719** | **0.339** | **0.018** | **0.230** | **-2.12** | **318** | **0.034** |
| # Leaves | 2.34 | 7.92 | <0.001 | 0.436 | 0.295 | 318 | 0.768 |
| SLA | 1.17 | 0.917 | 0.006 | 0.270 | 1.27 | 318 | 0.203 |
| LDMC | 10.7 | 9.18 | 0.005 | 0.330 | 1.17 | 318 | 0.243 |
| **LCC** | **-3.18** | **0.876** | **0.050** | **0.90** | **-3.63** | **318** | **<0.001** |
| LNC | 0.075 | 0.093 | 0.003 | 0.109 | 0.80 | 318 | 0.424 |
| **Understory** |  |  |  |  |  |  |  |
| Height | 0.015 | 0.056 | <0.001 | 0.216 | 0.261 | 101 | 0.793 |
| Stem Width | 0.989 | 0.602 | 0.022 | 0.227 | 1.64 | 101 | 0.100 |
| # Leaves | 9.96 | 5.25 | 0.030 | 0.221 | 1.90 | 101 | 0.058 |
| SLA | 2.48 | 2.24 | 0.011 | 0.179 | 1.10 | 101 | 0.270 |
| LDMC | 5.55 | 8.46 | 0.004 | 0.045 | 0.656 | 101 | 0.512 |
| LCC | -0.218 | 0.410 | 0.002 | 0.198 | -0.531 | 101 | 0.595 |
| LNC | 0.153 | 0.115 | 0.014 | 0.284 | 1.33 | 101 | 0.184 |

Table 3: Linear mixed effect models of CWM values in the Meadow and Understory habitats not including DSV trait values. Predictor variable was DSV relative abundance, site was included as a random effect.
